# Effect of Carboxymethylation and Phosphorylation on the Properties of Polysaccharides from *Sepia esculenta* Ink: Antioxidation and Anticoagulation In Vitro

**DOI:** 10.3390/md17110626

**Published:** 2019-11-01

**Authors:** Huazhong Liu, Fangping Li, Ping Luo

**Affiliations:** College of Chemistry & Environment, Guangdong Ocean University, Zhanjiang 524088, China; liuhzbs@163.com (H.L.); 15709482571@163.com (F.L.)

**Keywords:** *Sepia esculenta* ink polysaccharide, carboxymethylation, phosphorylation, antioxidation, anticoagulation

## Abstract

To investigate the effect of carboxymethylation and phosphorylation modification on *Sepia esculenta* ink polysaccharide (SIP) properties, this study prepared carboxymethyl SIP (CSIP) with the chloracetic acid method, and phosphorylated SIP (PSIP) with the sodium trimetaphosphate (STMP)/sodium tripolyphosphate (STPP) method, on the basis of an orthogonal experiment. The in vitro antioxidant and anticoagulant activities of the derivatives were determined by assessing the scavenging capacity of the 1,1-diphenyl-2-picrylhydrazyl (DPPH) and hydroxyl radicals, which activated the partial thromboplastin time (APTT), prothrombin time (PT), and thrombin time (TT). The results showed that SIP was modified successfully to be CSIP and PSIP, and degrees of substitution (DSs) of the two products were 0.9913 and 0.0828, respectively. Phosphorylation efficiently improved the antioxidant property of SIP, and the IC_50_ values of PSIP on DPPH and hydroxyl radicals decreased by 63.25% and 13.77%, respectively. But carboxymethylation reduced antioxidant activity of the native polysaccharide, IC_50_ values of CSIP on the DPPH and hydroxyl radicals increased by 16.74% and 6.89%, respectively. SIP significantly prolonged the APTT, PT, and TT in a dose-dependent fashion, suggesting that SIP played an anticoagulant action through intrinsic, extrinsic, and common coagulation pathways. CSIP and PSIP both possessed a stronger anticoagulant capacity than SIP via the same pathways; moreover, CSIP was observed to be more effective in prolonging APTT and PT than PSIP.

## 1. Introduction

*Sepia esculenta* ink polysaccharides (SIP) is a type of glycosaminoglycans derived from *Sepia esculenta* ink that has proven to be multifunctional in marine materials, displaying properties such as antioxidation, antitumor, immunoregulation, and chemoprevention [1,2]. Our previous work reported that SIP derived from *Sepia esculenta* ink is mainly composed of galactosamine and arabinose, as well as a small amount of fucose and other monosaccharides [3]. As SIP has been found to have properties of chemoprevention, antitumor, and chemosensitization, the natural polysaccharide was recognized as a potential adjuvant agent of chemotherapy [1]. It is well known that chemotherapy induces blood hypercoagulability, and the consequent thrombus formation is a critical cause of death in cancer cases [1]; therefore, whether SIP mediates blood coagulation is an unavoidable, crucial issue for developing the marine bioactive substance to be an adjuvant drug. Presently, no direct evidence shows an anticoagulation or procoagulation property of SIP. Sulphated SIP, a type of derivative of SIP isolated from *Ommastrephes bartrami* ink prepared by Chen et al., was found to possess an efficient anticoagulant activity, but the native SIP failed to alter the blood clotting time in vitro [4], suggesting that sulphation is an effective modification method for improving the property of SIP.

Chemical modification is well-recognized to be an effective way to change the physiochemical properties and to improve the biological activities of natural polysaccharides, which result from the alternation of the molecular structure caused by the conjugated functional groups [5]. It is a widely accepted notion that polysaccharide derivatives have a superior water solubility and antioxidant activity to native polysaccharides. Many modification methods have been widely used to modify native polysaccharides for improving properties, such as sulfation, methylation, hydroxypropylation, carboxymethylation, acetylation, phosphorylation, alkylation, and sulfonylation [6].

Hitherto, sulphated SIP is the only derivative of SIP, the native polysaccharide is derived from *Ommastrephes bartrami* ink. The modified polysaccharide shows anticoagulation, anti-angiogenesis, and anti-metastasis activities of HepG2 in tumor cells [4,7]. Another sulfated SIP prepared by sulfation on SIP isolated from *Sepiella maindroni* ink is anti-angiogenesis, as well as the anti-metastasis activities of SKOV3 tumor cells [8,9,10]. At present, no other derivatives of SIP have been reported, besides the sulphated SIP. To investigate the effect of the chemical modification on the SIP property, this study prepared carboxymethylated SIP (CSIP) and phosphorylated SIP (PSIP) to assess the effect of carboxymethylation and phosphorylation on SIP, based on antioxidation and anticoagulation.

## 2. Results

### 2.1. Determination of Carboxymethylation Parameters and Molecular Characteristics of CSIP

**Figure 1:**
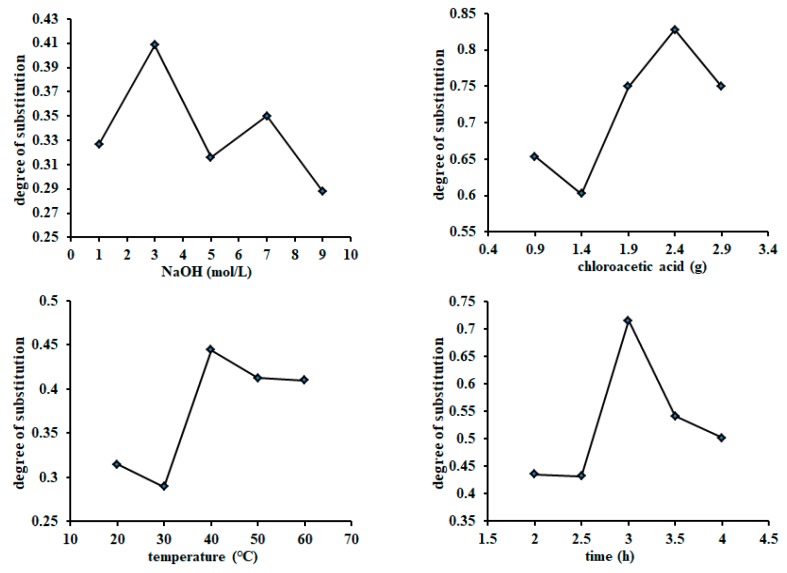
Effect of different reaction conditions on degree of substitution (DS) of carboxymethylated *Sepia esculenta* ink polysaccharides (CSIP). Under the scheduled different reaction conditions, the SIP was carboxymethylated by chloroacetic acid. The degree of substitution of CSIP was determined by the neutralization titration method and calculation according to the formula.

**Figure 2:**
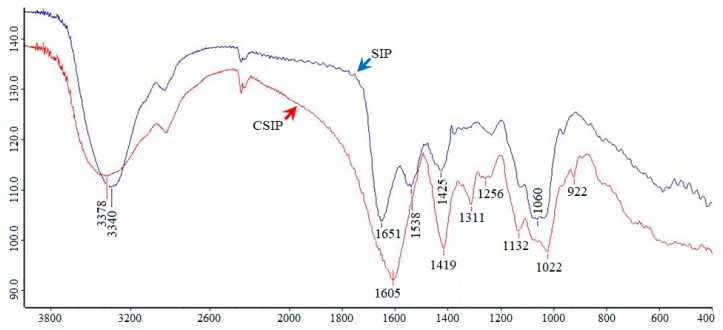
IR spectra of SIP and CSIP. IR spectra of the SIP and CSIP were recorded with KBr pellets on a Bruker Tensor 27 Fourier infrared spectrophotometer between 400–4000 cm^−1^.

The data presented in Figure 1 show that the four scheduled influence factors clearly affected the degree of substitution (DS) of the CSIP. The optimal factors were 3.0 mol/L (NaOH), 2.4 g (chloroacetic acid), 40 °C (reaction temperature), and 3 h (reaction time), respectively. The optimal combined parameters were 5.0 mol/L of NaOH solution, 2.84 g of chloroacetic acid, 50 °C, and 2.5 h, according to the values of *R* and DS (Table 1). The DS of the CSIP was calculated to be 0.9913. Different from the spectrum of the SIP, the CSIP spectrum presented two strong absorption peaks at 1605 and 1419 cm^−1^, two characteristic absorption bands of carboxymethyl of CSIP, suggesting that the SIP was carboxymethylated successfully (Figure 2).

### 2.2. Determination of the Phosphorylation Parameters and Molecular Characteristics of PSIP

**Figure 3:**
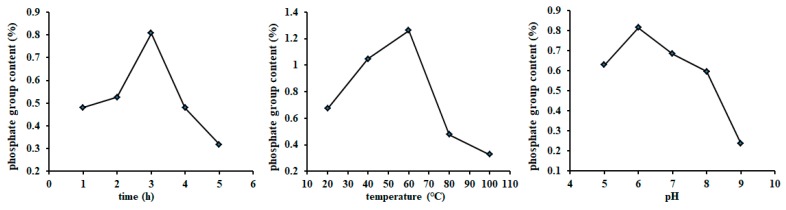
Effect of different reaction conditions on the content of phosphate in phosphorylated SIP (PSIP). Under the scheduled different reaction conditions, the SIP was phosphorylated with the STMP/STPP method. The content of phosphate in PSIP was determined according to the equation, which was subjected to calculating the degree of substitution of PSIP.

**Figure 4:**
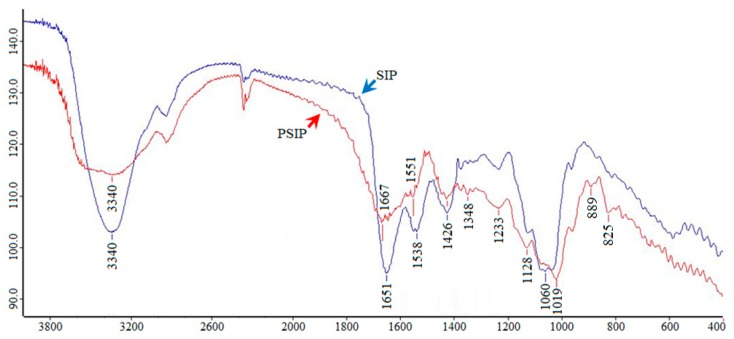
IR spectra of SIP and PSIP. The IR spectra of SIP and PSIP were recorded with KBr pellets on a BRUKER TENSOR 27 Fourier infrared spectrophotometer between 400–4000 cm^−1^.

A single factor experiment revealed that the optimal ratio of the sodium tripolyphosphate (STPP)/sodium trimetaphosphate (STMP), reaction time, temperature, and pH value was determined to be 50/20 (Table 2), 3.0 h, 40 °C, and pH 6.0, respectively, which were subjected to the orthogonal experiment (Figure 3). The optimal combined parameters were determined to be 40/30 (STPP/STMP), 3.0 h, 55 °C, and pH 5.0, respectively, according to the values of *R* and DS (Table 3). The phosphate content of PSIP was 1.52%, and the DS was calculated to be 0.0828. Compared with the spectrum of SIP, the PSIP spectrum observed two distinct absorption peaks at 1019 cm^−1^ (P–OH bond) and 889 cm^−1^ (P–O–C bond), suggesting that SIP was phosphated successfully (Figure 4).

### 2.3. Antioxidation Activity of CSIP and PSIP

**Figure 5:**
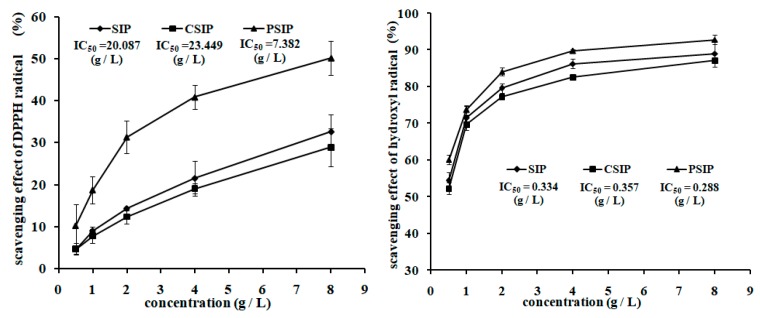
In vitro antioxidant activity of SIP, CSIP, and PSIP. The antioxidant capacity of SIP and its derivatives was assessed by the scavenging activities of DPPH and hydroxyl radicals.

Figure 5 presents that the scavenging activity of SIP on the 1,1-diphenyl-2-picrylhydrazyl (DPPH) radical was stronger than CSIP, but weaker than PSIP, which was demonstrated by the modified IC_50_ values—the PSIP dropped by 63.25% and the CSIP increased by 16.74%. Similarly, with respect to the scavenging capacity of SIP on the hydroxyl radical, the CSIP was weaker and the PSIP was stronger, and the IC_50_ values of the CSIP and PSIP increased by 6.89% and decreased by 13.77%, respectively. The data suggest that phosphorylation promoted the in vitro antioxidation activity of SIP, but carboxymethylation inhibited the property of SIP.

### 2.4. Anticoagulation Activity of CSIP and PSIP

**Figure 6:**
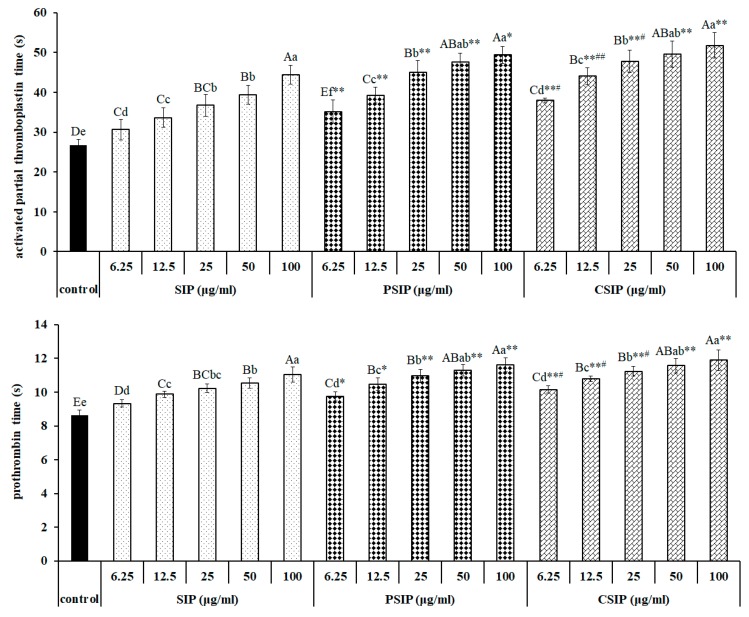

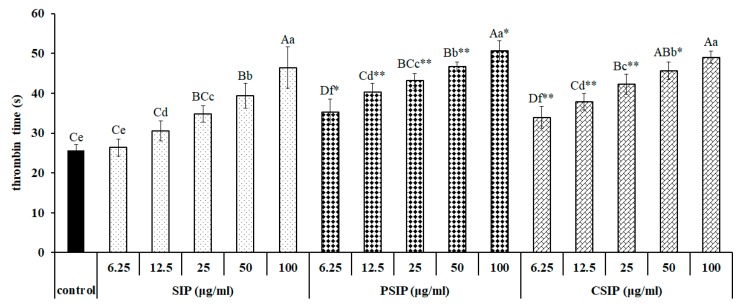
In vitro anticoagulant property of SIP, CSIP, and PSIP. The anticoagulant capacity of SIP and its derivatives was assessed by determining the clotting time, including the activated partial thromboplastin time (APTT), prothrombin time (PT), and thrombin time (TT). Different capital or lowercase letters express significant differences among various dosages (0, 6.25, 12.5, 25, 50, and 100) of polysaccharide (SIP, CSIP, or PSIP), ^ABCDE^ *p* < 0.01 or ^abcdef^ *p* < 0.05. Asterisk, * or **, means *p* < 0.05 or *p* < 0.01 between the same dosage of SIP and modified SIP (CSIP or PSIP), respectively. ^#^ or ^##^ represent the difference, *p* < 0.05 or *p* < 0.01, between the same dosage of CSIP and PSIP, respectively.

The activated partial thromboplastin time (APTT), prothrombin time (PT), and thrombin time (TT), three indicators evaluating the in vitro anticoagulation activity of the anticoagulant, express intrinsic, extrinsic, and common pathways of blood coagulation, respectively. The data in Figure 6 show that SIP prolonged the coagulation time in a dose-dependent manner, including APTT, PT, and TT, indicating that SIP had an anticoagulation action via endogenous, exogenous, and common coagulation pathways. Meanwhile, CSIP and PSIP both were observed in the capacity of extending the APTT, PT, and TT in dose-dependent fashions. Importantly, compared with SIP, both CSIP and PSIP showed a significantly stronger anticoagulant activity that was demonstrated by an extended APTT, PT, and TT. Moreover, CSIP was observed to have a stronger anticoagulant capacity than PSIP, ranging from 6.25 to 25 μg/mL, which was indicated by APTT and PT. These data indicate that SIP and its derivatives CSIP and PSIP, performed an anticoagulation action via intrinsic, extrinsic, and common pathways of blood coagulation. Moreover, both carboxymethylation and phosphorylation promoted the activity of the native polysaccharide, and the promotion of carboxymethylation was slightly superior to the phosphorylation modification.

## 3. Discussion

Carboxymethylation and phosphorylation have been widely used to modify natural polysaccharides for improving the physiochemical and biological properties of the native macromolecules [11]. In the present study, SIP was modified with chloroacetic acid and STMP/STPP methods, respectively. Through the chemical modification methods, SIP was successfully modified to be CSIP and PSIP, and their DSs were determined to be 0.9913 and 0.0828, respectively. DS is an important influencing factor of the biological activity of the polysaccharide derivative, indicating that CSIP and PSIP may possess different properties from SIP. 

Currently, three types of SIPs have been reported, including polysaccharides from *Ommastrephes bartrami* ink, *Illex argentinus* ink, *Sepiella maindroni* ink, and *Sepia esculenta* ink. The first two SIPs share the same primary structure [1]. No evidence indicated a group of carboxymethyl or phosphate in SIP. The SIP used in this study was derived from *Sepia esculenta* ink, and was mainly composed of galactosamine and arabinose (molar ratio of 1:1), accounting for more than 81% of monosaccharides. In addition, fucose, xylose, glucuronic acid, galacturonic acid, glucosamine, and mannose were about 9.00%, 4.32%, 1.98%, 1.53%, 1.35%, and 0.09%, respectively [3]. Both the carboxymethylation and phosphorlation reactions occur on the hydroxyl group of polysaccharides, therefore, it is still unable to confirm which sites are substituted by carboxymethyl or phosphate until the primary structures of SIP, CSIP, and PSIP have been characterized.

The carboxymethyl group is an electron-withdrawing group that can influence the antioxidant capacity of heterocyclic compounds, by increasing or decreasing [12,13]. The opposite effect of carboxymethylation on natural polysaccharides has been proven by many reports [11,12,14,15,16,17,18]. This work exhibited a negative effect of carboxymethylation on the SIP antioxidant property. As the molecular structure of the SIP used in this study is still unknown, the involved mechanism of carboxymethylation on the antioxidant ability of SIP is unable to be explained. 

Phosphorylation improving the antioxidant activity of polysaccharides result from the conjugated phosphate groups via ester linkage [19], which is partly attributed to phosphate DS, a critical influencing factor [20]. Phosphoryl polysaccharide generally possesses a low DS, and a stronger bioactivity than the sulfated derivative [21,22,23]. Our data revealed that the DS of PSIP was 0.0828. The DS of phosphorylated polysaccharide has a close relationship with the phosphorylation methods. Many methods have been developed in order to prepare phosphorylated polysaccharides [24,25]. The present work prepared PSIP with the STMP method. The mechanism involved in the synthesis of PSIP is still unclear, but it can probably be deduced according to the findings [25]. STMP is a cross-linking agent that triggers a reaction between the hydroxyl groups of SIPs and the metaphosphate groups in STMP, resulting in phosphate ester (O–P–O) linkages between two polysaccharide moieties.

Three negative charges of the phosphate radical result in an increase of electronegativity and activity alternation of the polysaccharide. The phosphorylation modification improved the in vitro antioxidant activity of the SIP, demonstrated by promoted the scavenging capacity of the DPPH and hydroxyl radicals. Similar results were found in garlic polysaccharide [6], *Enteromorpha linza* polysaccharide [26], *Dictyophora indusiata* polysaccharide [24], *Portulaca oleracea* L. polysaccharide [20], pumpkin polysaccharide [27], and native ginseng polysaccharide [28].

The SIP from *Ommastrephes bartrami* ink is ineffective at changing the blood coagulation time in vitro [7]. However, the SIP from *Sepia esculenta* ink was proven to have an obvious anticoagulant activity in this work. This type of SIP is a kind of sulfate polysaccharide with a 2.25% sulfate content, but the sulfate was not detected in the SIP from *Ommastrephes bartrami* ink. Generally, the anticoagulant property of the sulfate polysaccharide is related to the sulfate content [29]. Moreover, the sulfated SIP (molar ratio of monosaccharide/sulfate group: 3/2) markedly extended the APTT and PT via inhibiting the blood coagulation factors IIa and Xa [7]. So, one important cause of the anticoagulant property should be the existence of sulfate in SIP used in this study. Another crucial cause may be the molecular structure—the two types of SIPs possess entirely different primary structures. 

It is widely accepted that chemical modification effectively improves the physicochemical and biological properties of natural polysaccharides. This work testified that carboxymethylation and phosphorylation high-effectively improved the anticoagulant activity of SIP, indicated by prolonged APTT, PT, and TT. Interestingly, carboxymethylation was more effective in improving the anticoagulant capacity of SIP than phosphorylation.

Various phosphorylated polyanions play an anticoagulant action through enhancing the complexation of heparin cofactor II and thrombin [30], the binding is mainly related to the negative charges and the polymeric nature [31]. The phosphorylation increasing anticoagulant property has been found in oat spelts’ xylan [29], which were related to the molecular weight (positively) and phosphate content (inversely) [31], as well as the highly polymeric nature, and phosphodiester or diphosphodiester bonds [29].

Currently, there is little understanding of the molecular structure of the novel SIP and its derivatives, CSIP and PSIP, and the structure-activity relationship needs to be further investigated in order to explain the involved regulatory mechanisms of carboxymethylation, phosphorylation on antioxidation, and the anticoagulation of SIP.

## 4. Materials and Methods

### 4.1. Preparation of SIP

According to the reported method of Gu et al. [32], the procedure is briefly described as follows. Fresh *Sepia esculenta* ink stored at −70 °C was thawed at 4 °C, diluted in phosphate buffer solution, and then ultrasonicated. After storage at 4 °C for more than 8 h and then centrifugation (8000 rpm) at 4 °C, the supernatant was hydrolyzed with papain for 90 min, and then heated for 1 h in a boiling water bath. Following centrifugation, four volumes of ethanol were used to precipitate the crude polysaccharides that were then subjected to DEAE-52 cellulose column chromatography. The first fraction containing SIP was collected and dialyzed, desiccated in a vacuum freeze drier, and stored at −20 °C.

### 4.2. Preparation of CSIP

An orthogonal experimental design was used to prepare the CSIP. The SIP (100 mg) was dissolved in 20 mL of NaOH solution (1.0, 3.0, 5.0, 7.0, and 9.0 mol/L), and stirred for 30 min at room temperature. Chloracetic acid (0.95, 1.42, 1.89, 2.37 and 2.84 g) was dissolved in a SIP solution and heated in a water bath (20, 30, 40, 50, and 60 °C) for a scheduled time (2.0, 2.5, 3.0, 3.5, and 4.0 h). The reaction system was cooled to room temperature and neutralized to pH 7.0 with glacial acetic acid. After dialysis against distilled water for 72 h, the solution was dried at 75 °C in a drier to harvest the CSIP.

The DS was determined according to the method of neutralization titration, with slight modification [33]. The CSIP sample (10 mg) dissolved in 3 mL of methanol (70%) was added in 15 mL of NaOH solution (0.167 mol/L). Immediately, the mixture was titrated with an HCl solution (0.1 mol/L). The DS was calculated by the following formulas:

*A* = (*V*_0_*M*_0_ − *VM* )/*W*,

DS = 0.162 *A*/(1 − 0.058*A*),

where *V_0_* = 5 mL; *M_0_* = 0.5 mol/L; *M* = 0.1 mol/L; and *V* and *W* represent the consumed volume of the hydrochloric acid (mL) and sample weight (g), respectively.

### 4.3. Preparation of PSIP

An orthogonal experimental design was used to prepare the PSIP. The SIP solution (10 mL) in distilled water (10 mg/mL) was added in 4.285 mL of a mixed solution (70 mg/mL) containing STPP and STMP, and 0.2 mL of NaSO_4_ solution (50 mg/mL). The contents of STPP and STMP in the mixed solution are presented in Table 2. The reaction system was neutralized to scheduled pH values (5.0, 6.0, 7.0, 8.0, and 9.0) with HCl (1.0 mol/L), and cooled to scheduled temperatures (25, 45, 65, 85, and 100 °C). After reaction for a scheduled time (2.0, 3.0, 4.0, 5.0, and 6.0 h), three volumes of ethanol were used to precipitate the product for 24 h at room temperature. The precipitate was dried at 75 °C in a drier, and then dissolved in 15 mL of distilled water at 50 °C in a water bath. The conductivity determination of the solution occurred in the process of dialysis against distilled water. The dialysis was stopped when the conductivity declined to be 160 μs/cm. The solution was dried at 75 °C in a drier so as to harvest PSIP.

The phosphate content was determined according to the reported method, with slight modification; the standard curve of the phosphate radical was prepared according to the procedure [34]. Different volumes (0, 0.5, 1.0, 1.5, 2.0, 2.5, 3.0, 3.5, 4.0, 4.5, and 5.0 mL) of a phosphate standard solution (0.1 mg/mL) were mixed with distilled water, respectively, and the total volume reached 5.0 mL. Tris buffer solution (3.0 mL, 0.1 mol/L, pH 7.0) was added into 3 mL of phosphate quantitative reagent, reacting for 30 min at a temperature of 45 °C. Then, the absorbance was read at a wavelength of 580 nm. The standard curve of the phosphate radical content was established with the absorbance (ordinate) and phosphate radical content (abscissa). The phosphate content in the PSIP was determined according to the method in the literature [35]. The dried PSIP (10 mg) was ashed for 2 h at 600 °C, and then dissolved in 0.5 mL of HCl solution (0.5 mol/L), and diluted with distilled water to 10 mL. The ashed compound solution (1.0 mL) was put into a 25 mL cuvette, and the absorbance was read according to the abovementioned standard curve method. Based on the standard curve, phosphate radical content in the PSIP was calculated. 

### 4.4. Infrared Spectral Analysis

The IR spectra of the SIP, CSIP, and PSIP were recorded with KBr pellets on a Bruker Tensor 27 Fourier infrared spectrophotometer (Bruker, Karlsruhe, Germany) between 400–4000 cm^−1^. 

### 4.5. Scavenging Activity of Hydroxyl Radical

The scavenging activity of the hydroxyl radical was assessed according to the method in the literature, with slight modification [33]. A sample (SIP, CSIP, or PSIP) was dissolved in distilled water, to be different concentrations, namely, 0.5, 1.0, 2.0, 4.0, and 8.0 g/L. The sample solution (1.0 mL) was diluted in 2.0 mL of a phosphate buffer solution (0.02 mol/L, pH 7.4), and was mixed with 1,10-phenanthroline (1.5 mL, 1.0 mmol/L), FeSO_4_ (1.0 mL, 1.5 mmol/L), H_2_O_2_ (1.0 mL, 1.0%), and distilled water (3.5 mL). Following incubation for 60 min at 37 °C, the absorbance was read at a wavelength of 536 nm. The scavenging activity was calculated according to the following formula:

$$\mathrm{Scavenging}\mathrm{activity}(\%)=\frac{OD_{2}-OD_{1}}{OD_{0}-OD_{1}}\times 100\%,$$

where the sample was replaced by H_2_O in the OD_1_ associated reaction system, H_2_O replaced the sample and H_2_O_2_ in the OD_0_ associated reaction system, and OD_2_ associated with the reaction system contained the sample. 

### 4.6. Scavenging Activity of DPPH

The scavenging activity of the DPPH radical was determined according to the method from the literature, with slight modification [33]. A DPPH solution (2.0 mL, 0.1 mmol/L) was mixed with 0.5 mL of different concentrations of SIP, CSIP, or PSIP (0.5, 1.0, 2.0, 4.0, and 8.0 g/L), and 1.5 mL of distilled water, and was then kept away from light for 30 min at room temperature. The absorbance was read at a wavelength of 517 nm. The scavenging activity was calculated as the following formula.

$$\mathrm{Scavenging}\mathrm{activity}(\%)=\frac{1-(OD_{2}-OD_{1})}{OD_{0}}\times 100\%$$

where OD_0_ represents the negative control that is composed by 2.0 mL DPPH and 2.0 mL ethanol. OD_1_ is a mixture of 2.0 mL ethanol, 0.5 mL of the sample solution, and 1.5 mL distilled water. OD_2_ consists of 2.0 mL of an DPPH–ethanol solution, 0.5 mL of a sample solution, and 1.5 mL distilled water. 

### 4.7. Determination of the Anticoagulation Activity

Male Kunming mice (20–25 g) purchased from Guangdong Medical Animal Experiment Center (SCKY-2018-0002) were habituated for 1 week and were randomly allocated to six groups, control group and five treated groups, ten mice in each group. Mice were sacrificed to harvest blood that was then prepared serum immediately. Each group of murine sera was treated with distilled water or different dosages (6.25, 12.5, 25, 50 and 100 μg/mL) of SIP, CSIP or PSIP solution in distilled water and then subjected to determine APTT, PT and TT. 

The activated partial thromboplastin time (APTT), prothrombin time (PT) and thrombin time (TT) of the SIP, CSIP, and PSIP were assessed with kits, purchased from Nanjing Jiancheng Bioengineering Institute (Nanjing, China), according to the manufacturer’s protocols. 

### 4.8. Statistical Analysis

The data were expressed as mean ± the standard error, and were analyzed using SPSS statistical software. One-way analysis of variance (ANOVA) of the data was performed. *p* < 0.05 was considered to be a significant level.

## 5. Conclusions

In summary, the SIP was successfully modified to be CSIP with the chloracetic acid method, and to be PSIP with the STMP/STPP method, on the basis of the orthogonal experiment. The DSs of the two products were 0.9913 and 0.0828, respectively. The results revealed that the phosphorylation efficiently improved the antioxidant property of the SIP, but carboxymethylation reduced the activity of the native polysaccharide. Additionally, we found a strong in vitro anticoagulant action of SIP from *Sepia esculenta* ink via intrinsic, extrinsic, and common coagulation pathways, which was different from the SIP from *Ommastrephes bartrami* ink that did not observe an anticoagulant activity [4]. Moreover, the phosphorylation and carboxymethylation both could enhance the anticoagulant capacity of SIP, and CSIP showed a more effective anticoagulation action than PSIP in intrinsic and extrinsic pathways, but no obvious difference between them was observed in the common pathway.

## Figures and Tables

**Table 1:** Orthogonal experimental design and results of the SIP carboxymethylation.

Items	Chloroacetic Acid (g)	NaOH (mol/L)	Temperature (℃)	Time (h)	DS
1	1.89	1.0	30	2.5	0.43
2	1.89	3.0	40	3.0	0.60
3	1.89	5.0	50	3.5	0.58
4	2.37	1.0	40	3.5	0.45
5	2.37	3.0	50	2.5	0.75
6	2.37	5.0	30	3.0	0.75
7	2.84	1.0	50	3.0	0.61
8	2.84	3.0	30	3.5	0.67
9	2.84	5.0	40	2.5	0.84
*K_1_*	0.49	0.53	0.61	0.67	
*K_2_*	0.67	0.65	0.63	0.65	
*K_3_*	0.72	0.70	0.64	0.56	
*R*	0.23	0.17	0.03	0.11	

**Table 2:** Effect of the phosphorylating reagent on the content of phosphate. STPP—sodium tripolyphosphate; STMP—sodium trimetaphosphate.

Items	STPP (g/L)	STMP (g/L)	Phosphate Radical Content (%)
1	0	0	0.305
2	70	0	1.451
3	60	10	2.095
4	50	20	3.818
5	40	30	3.661
6	30	40	2.826
7	20	50	3.081
8	10	60	1.921
9	0	70	2.269

**Table 3:** Orthogonal experimental design and results of SIP phosphorylation.

Items	STPP/STMP (g/L)	Temperature (°C)	Time (h)	pH	Phosphate Content (%)
1	40/30	55	3	5.0	1.52
2	40/30	65	4	6.0	0.33
3	40/30	75	5	7.0	0.63
4	50/20	55	4	7.0	0.20
5	50/20	65	5	5.0	0.76
6	50/20	75	3	6.0	0.39
7	60/10	55	5	6.0	0.52
8	60/10	65	3	7.0	0.55
9	60/10	75	4	5.0	0.25
*K_1_*	0.82	0.74	0.82	0.84	
*K_2_*	0.45	0.54	0.26	0.41	
*K_3_*	0.44	0.42	0.63	0.46	
*R*	0.38	0.32	0.56	0.43	
